# Sexual health norms and communication patterns within the close social networks of men who have sex with men and transgender women in Lima, Peru: a 2017 cross-sectional study

**DOI:** 10.1186/s12889-021-11091-2

**Published:** 2021-06-07

**Authors:** Amrita Ayer, Eddy R. Segura, Amaya Perez-Brumer, Susan Chavez-Gomez, Rosario Fernandez, Jessica Gutierrez, Karla Suárez, Jordan E. Lake, Jesse L. Clark, Robinson Cabello

**Affiliations:** 1 South American Program in HIV Prevention Research, University of California Los Angeles David Geffen School of Medicine, 10833 Le Conte Avenue, CHS 37-121, Los Angeles, CA 90095 USA; 2 University of California Los Angeles David Geffen School of Medicine, Los Angeles, USA; 3 Escuela de Medicina, Universidad Peruana de Ciencias Aplicadas, Lima, Peru; 4 University of Toronto Dalla Lana School of Public Health, Toronto, Canada; 5 Asociación Civil Via Libre, Lima, Peru; 6 Department of Internal Medicine, Division of Infectious Diseases, McGovern Medical School at UT Health, Houston, USA; 7 Department of Internal Medicine, Division of Infectious Diseases, David Geffen School of Medicine at UCLA, Los Angeles, USA

**Keywords:** Men who have sex with men (MSM), Transgender women (trans women), Sexually transmitted infections (STIs), HIV prevention, Social networks, Peru

## Abstract

**Background:**

Social networks, norms, and discussions about sexual health may inform sexual practices, influencing risk of human immunodeficiency virus (HIV) or sexually transmitted infection (STI) acquisition. To better understand social networks of Peruvian men who have sex with men (MSM) and transgender women (trans women), we examined key social network members (SNMs), participant perceptions of these network members’ opinions toward sexual health behaviors, and associations between network member characteristics and condomless anal intercourse (CAI).

**Methods:**

In a 2017 cross-sectional study, a convenience sample of 565 MSM and trans women with HIV-negative or unknown serostatus was asked to identify three close SNMs; describe discussions about HIV and STI prevention with each; and report perceived opinions of condom use, HIV/STI testing, and partner notification of STIs. Generalized estimating equations evaluated relationships between SNM characteristics, opinions, and discussions and participant-reported CAI.

**Results:**

Among participants who identified as MSM, 42.3% of key SNMs were perceived to identify as gay. MSM “never” discussed HIV and STI prevention concerns with 42.4% of heterosexual SNMs, but discussed them “at least once weekly” with 16.9 and 16.6% of gay- and bisexual- identifying SNMs, respectively. Among participants who identified as trans women, 28.2% of key SNMs were perceived as heterosexual; 25.9%, as bisexual; 24.7%, as transgender; and 21.2%, as gay. Trans women discussed HIV/STI prevention least with cis-gender heterosexual network members (40.2% “never”) and most with transgender network members (27.1% “at least once weekly”). Participants perceived most of their close social network to be completely in favor of condom use (71.2% MSM SNMs, 61.5% trans women SNMs) and HIV/STI testing (73.1% MSM SNMs, 75.6% trans women SNMs), but described less support for partner STI notification (33.4% MSM SNMs, 37.4% trans women SNMs). Most participants reported CAI with at least one of their past three sexual partners (77.5% MSM, 62.8% trans women). SNM characteristics were not significantly associated with participant-reported frequency of CAI.

**Conclusions:**

Findings compare social support, perceived social norms, and discussion patterns of Peruvian MSM and trans women, offering insight into social contexts and sexual behaviors.

**Trial registration:**

The parent study from which this analysis was derived was registered at ClinicalTrials.gov (Identifier: NCT03010020) on January 4, 2017.

## Introduction

Social networks can shape sexual practices and alter risks for the acquisition of human immunodeficiency virus (HIV) and other sexually transmitted infections (STIs) [1–4]. Social network studies have been employed in diverse global settings to better understand sexual health behaviors and disease transmission patterns and direct prevention efforts among groups experiencing high burdens of HIV and STIs, including men who have sex with men (MSM) and transgender women (trans women) [1, 4–6].

Both network structures and characteristics contribute to the relationship between social networks and sexual practices, in part through the construction of shared social environments and peer norms [1, 4]. For example, network size, density, and composition have been shown to relate to condom use in intercourse and other sexual practices [1, 3, 7–9], although there may be variability in these relationships with context or identity [10]. Similarly, social norms may influence sexual behaviors: In a study of Black and Latino MSM in the United States, low levels of social support for condom use were associated with increased report of condomless anal intercourse (CAI) [7]. Other studies have supported the link between peer norms and sexual practices [1, 2, 11, 12], but relationship characteristics may modify network impact. Closeness of relationships between social contacts may affect perceptions of social norms and/or potentiate the interaction between these norms and safer sexual practices [3, 13, 14].

Within Latin America, prior research has described the ways in which social networks serve as sources of support, information, and influence among MSM and trans women [15, 16]. Social and sexual interactions may serve to disseminate knowledge and resources about HIV or STI prevention through MSM and trans women’s networks, where gender identity, sexual orientation, and sexual role may influence awareness of and engagement with HIV/STI prevention resources [16, 17]. Network recruitment patterns from a study in El Salvador and results from a pilot trial of a social network-based pre-exposure prophylaxis (PrEP) intervention in Peru suggest that trans women’s social networks may play important roles in HIV prevention [18, 19]. However, other literature points to the need to consider social relationship characteristics and individual or peer identities in network dynamics, communication patterns, and relationship to sexual health. Gender and sexual identity, sexual role, and relationship type may influence peer discussions about topics such as HIV [3, 15–17]. The effect of close contacts’ opinions about sexual health may in turn vary with patterns of communication, as well as broader network and partnership contexts [20].

These works offer insight into social network dynamics of MSM and trans women in Latin America and their potential role in HIV and STI prevention efforts. However, there is still a dearth of information delineating social network structures and evaluating the connections of these network characteristics with sexual health behaviors in these contexts. Given that Peruvian MSM and trans women are disproportionately affected by HIV and STIs [21, 22], we sought to address this gap by studying social networks, norms, and sexual practices of a cohort of MSM and trans women reporting recent receptive CAI in Lima, Peru.

Prior research with this cohort has suggested that CAI is frequent and sexual communication with partners, limited [23]; thus, understanding the social environments experienced by these communities may provide insight into positive and negative influences on sexual behavior and opportunities for intervention [15]. In this analysis, we aimed to characterize key social network members (SNMs) within the social networks of Peruvian MSM and trans women; explore perceptions of SNM opinions toward sexual health behaviors; and examine associations of SNM attitudes and characteristics with CAI.

## Methods

### Study population

We analyzed screening visit data from a 2017 study of rectal STI screening and bio-behavioral HIV prevention. MSM and trans women were recruited from community venues in Lima, Peru. Eligibility included: 1) age ≥ 18 years; 2) male sex assigned at birth; 3) self-reported HIV-negative serostatus; and 4) condomless receptive anal intercourse (CRAI) with an HIV-positive or unknown serostatus partner in the previous 6 months. All participants provided written informed consent prior to enrollment. The study was approved by the Institutional Review Boards of the University of California, Los Angeles and *Asociación Civil Vía Libre.*

### Procedures

Full study procedures have previously been described [24]. Participants completed a socio-behavioral survey via computer-assisted self-interview, underwent a physical exam, and received HIV and STI counseling and testing. Participants received 15 *Nuevos soles* (approximately $5.00), as well as five condoms and packets of lubricant.

### Measures

#### Participant and partner characteristics

We assessed participant demographics, sexual orientation/gender identity (heterosexual, gay, bisexual, or transgender), and sexual role during intercourse (*activo* [insertive], *moderno* [versatile], or *pasivo* [receptive]). Additionally, we collected information about participants’ previous three sexual partnerships, including relationship type (dichotomized as transactional or non-transactional), alcohol use prior to intercourse, and condomless receptive or insertive anal intercourse (combined into a single variable, CAI). All partnership-specific questions were defined at the participant-level as occurring with *any* of the previous three sexual contacts.

#### SNM characteristics

Participants were asked to identify a maximum of three close social network members (“*personas influyentes*” or “influential people”) to whom they had been close in the past 3 months. They characterized each SNM as a parent, sibling, cousin/relative, partner, friend, or colleague and described their perception of the SNM’s sexual orientation/gender identity. We measured frequency of participant/SNM discussions regarding “questions or concerns about HIV or STI prevention.” Five-point Likert scales (Completely Opposed – Completely in Favor) assessed participant perceptions of SNM opinions toward regular HIV and STI testing, condom use for HIV prevention, and partner notification of an STI diagnosis. We recategorized SNM relationships (“family, partner, friend, or colleague”) and discussions of HIV/STI prevention concerns (“never” versus “ever”) to evaluate associations with participant-reported CAI.

#### Social and sexual network characteristics

Participants estimated the number of lesbian, gay, bisexual, and transgender (LGBT) contacts in their close and overall social networks and described their sexual network size (number of distinct sexual contacts) over the past 30 days.

### Data analysis

We restricted analysis to participants reporting at least one SNM and providing data on recent sexual partners. Since screening eligibility criteria included recent CRAI, few participants identified their sexual role during intercourse as *activo* (insertive), and we further limited inclusion to individuals reporting *pasivo* (receptive) or *moderno* (versatile) sexual roles.

All analyses were stratified by participant-reported gender identity (cisgender man or transgender woman). Descriptive statistics were calculated for participant, network, partner, and SNM characteristics. Mann-Whitney/Wilcoxon tests compared MSM and trans women’s networks. To account for clustering of SNM characteristics reported by the same participant, we calculated prevalence ratios using generalized estimating equations (GEE) with an independent working correlation structure [25, 26]. GEE tested associations of participant, network, partner, and SNM factors with the primary outcome: participant-reported CAI in any of the previous three sexual encounters. The outcome was constructed from responses to questions assessing sexual practices and use of condoms in each of the past three sexual encounters. Covariates for multivariate analyses were selected based on conceptual reasoning or statistical significance in crude analyses (*p* < 0.05). Analysis was conducted with Stata 14.2 (Stata Corp, College Town, TX).

## Results

Of 614 participants screened, 444 MSM and 121 trans women were eligible for analysis. MSM were a median age of 27 years (interquartile range (IQR 22–34) and, trans women, 29 years (IQR 24–38). The majority of participants identified their sexual orientation as gay (86.2% of MSM; 76.3% of trans women). Regarding educational attainment, most MSM reported greater than high school education (59.9%) while most trans women described completing high school (57.9%). Trans women described larger gay and bisexual social networks than MSM (trans women median 50, IQR 20–100; MSM median 30, IQR 15–100; *p* = 0.03) and reported more sexual contacts over the past 30 days (trans women median 10, IQR 4–11; MSM median 5, IQR 2–10; *p* < 0.001).

Table 1 presents participant, SNM, and partner characteristics and associations with participant-reported CAI in any of the past three sexual encounters. MSM reported 1332 close social network members. Among SNMs of MSM, 42.3% were perceived to identify as gay. MSM “never” discussed questions or concerns about HIV/STI prevention with 34.1% of SNMs. Discussions were least frequent with heterosexual SNMs (43.4% “never” discussed) and most frequent (“at least once weekly”) with gay- (16.9%) and bisexual- (16.6%) identifying SNMs. MSM reported most SNMs to be completely in favor of condom use (71.2%) and HIV/STI testing (73.1%), but perceived lower support for disclosing an STI diagnosis to a partner (33.4% completely in favor). Among different SNM types, sexual partners were described as most supportive of condom use by MSM (17.7% completely in favor). Regarding sexual practices, 77.5% of MSM reported CAI with any of their previous 3 partners.

**Table 1:** Participant, SNM, and partner characteristics and associations with CAI among Peruvian MSM and Trans Women, 2017

	MSM			Trans Women		
	Median or *N* (IQR or %)	PR (95% CI)	aPR (95% CI)	Median or N (IQR or %)	PR (95% CI)	aPR (95% CI)
PARTICIPANT	***N*** **= 444**			***N*** **= 121**		
*Age* (*n* = 443 MSM, 121 Trans Women)	27 (22,34)	1.00 (0.99, 1.00)	1.00 (0.99, 1.00)	29 (24,38)	1.01 (1.00, 1.02)	1.00 (0.99, 1.02)
*Sexual Role* (*n* = 437 MSM, 121 Trans Women)						
*Pasivo* (receptive)	208 (47.6)	Ref.	Ref.	104 (85.9)	Ref.	Ref.
*Moderno* (versatile)	229 (52.4)	0.97 (0.88, 1.08)	1.00 (0.90, 1.09)	17 (14.1)	1.04 (0.71, 1.52)	1.06 (0.73, 1.54)
*Gender Identity/Sexual Orientation* (*n* = 437 MSM, 118 Trans Women)						
Heterosexual	6 (1.4)	Ref.	Ref.	8 (6.8)	Ref.	Ref.
Bisexual	69 (15.8)	1.02 (0.57, 1.84)	1.02 (0.57, 1.84)	6 (5.1)	0.67 (0.18, 2.53)	0.72 (0.24, 2.17)
Gay	362 (82.8)	1.19 (0.68, 2.11)	1.18 (0.67, 2.06)	90 (76.3)	1.31 (0.64, 2.67)	1.41 (0.76, 2.60)
Transsexual/ transgender	0			14 (11.8)	1.14 (0.50, 2.63)	1.18 (0.56, 2.47)
*Education*						
< High School	27 (6.1)	Ref.	Ref.	23 (19.0)	Ref.	Ref.
High School	151 (34.0)	1.07 (0.83, 1.39)	1.08 (0.83, 1.39)	70 (57.9)	1.41 (0.85, 2.34)	1.42 (0.86, 2.35)
> High School	266 (59.9)	1.13 (0.88, 1.45)	1.14 (0.89, 1.46)	28 (23.1)	**1.89 (1.15, 3.11)**	**1.87 (1.13, 3.10)**
Social Network Size						
*Number of Gay/Bisexual Contacts* (*n* = 389 MSM, 96 Trans Women) [ref.: < median]	30 (15,100)	0.98 (0.88, 1.09)		50 (20,100)	1.01 (0.73, 1.39)	
*Number of Gay/Bisexual Close Contacts* (*n* = 428 MSM, 112 Trans Women) [ref.: < median]	1 (1,3)	1.10 (1.00, 1.22)		1 (1,3)	1.13 (0.85, 1.50)	
*Sexual Network Size in the past 30 days* (n = 443 MSM, 121 Trans Women) [ref.: < median]	5 (2,10)	1.08 (0.98, 1.19)		10 (4,11)	0.80 (0.61, 1.05)	
KEY SOCIAL INFLUENCERS	***N*** **= 1332**			***N*** **= 363**		
*Sexual Orientation/Gender Identity* (*n* = 1286 MSM, 344 Trans Women)						
Heterosexual	433 (33.7)	Ref.		97 (28.2)	Ref.	
Bisexual	247 (19.2)	1.04 (0.95, 1.15)		89 (25.9)	0.83 (0.62, 1.11)	
Gay	544 (42.3)	1.05 (0.96, 1.14)		73 (21.2)	0.93 (0.72, 1.20)	
Transgender/sexual	62 (4.8)	0.98 (0.80, 1.19)		85 (24.7)	0.77 (0.56, 1.06)	
*Relationship with Participant* ^*a*^ (*n* = 1297 MSM, 355 Trans Women)						
Family		Ref.			Ref.	
Parent	104 (8.0)	–		20 (5.6)	–	
Sibling	68 (5.2)	–		30 (8.5)	–	
Cousin/other relative	56 (4.3)	–		14 (3.9)	–	
Spouse or partner	51 (3.9)	0.97 (0.81, 1.16)		25 (7.0)	1.21 (0.82, 1.77)	
Friend	963 (74.3)	1.02 (0.92, 1.13)		240 (67.6)	1.06 (0.79, 1.43)	
Colleague	55 (4.3)	0.95 (0.78, 1.15)		26 (7.3)	1.23 (0.85, 1.78)	
*Discussions of HIV “in addition to testing or prevention”* (*n* = 1319 MSM, 357 Trans Women) [ref.: no]	363 (27.5)	1.00 (0.91, 1.09)		77 (21.6)	0.90 (0.67, 1.20)	
*Freq. of discussion about HIV/STI questions or concerns* ^*b*^*:* (n = 1319 MSM, 357 Trans Women)						
Never	450 (34.1)	Ref.	Ref.	107 (30.0)	Ref.	
Ever		**1.11 (1.01, 1.22)**	1.08 (0.99, 1.19)		1.14 (0.87, 1.49)	
1x every few months	472 (35.8)	–	–	121 (33.9)	–	
1x every few weeks	209 (15.8)	–	–	58 (16.2)	–	
**≥** 1x/week	188 (14.3)	–	–	71 (19.9)	–	
Participant perceptions of KSI attitudes toward						
*Condom use for the prevention of HIV:* (*n* = 1317 MSM, 356 Trans Women)						
No Opinion	124 (9.4)	Ref.		49 (13.8)	Ref.	Ref.
Completely Opposed	11 (0.8)	1.20 (0.96, 1.50)		6 (1.7)	1.28 (0.80, 2.03)	1.57 (0.95, 2.57)
Partially Opposed	37 (2.8)	1.14 (0.95, 1.37)		8 (2.2)	**1.53 (1.14, 2.06)**	1.14 (0.82, 1.58)
Partially in Favor	208 (15.8)	1.03 (0.87, 1.22)		74 (20.8)	0.99 (0.69, 1.44)	0.93 (0.65, 1.34)
Completely in Favor	937 (71.2)	1.02 (0.87, 1.19)		219 (61.5)	0.94 (0.67, 1.30)	0.90 (0.65, 1.23)
*Disclosing an STI diagnosis to a partner:* (*n* = 1318 MSM, 356 Trans Women)						
No Opinion	348 (26.4)	Ref.		63 (17.7)	Ref.	
Completely Opposed	159 (12.1)	0.96 (0.83, 1.10)		35 (9.8)	0.90 (0.55, 1.48)	
Partially Opposed	90 (6.8)	0.95 (0.81, 1.13)		41 (11.5)	1.02 (0.68, 1.55)	
Partially in Favor	281 (21.3)	0.93 (0.83, 1.04)		84 (23.6)	0.93 (0.66, 1.31)	
Completely in Favor	440 (33.4)	0.92 (0.82, 1.03)		133 (37.4)	0.94 (0.69, 1.29)	
*HIV/STI Testing:* (*n* = 1319 MSM, 356 Trans Women)						
No Opinion	163 (12.3)	Ref.		33 (9.3)	Ref.	
Completely Opposed	9 (0.7)	0.54 (0.24, 1.21)		5 (1.4)	1.02 (0.58, 1.79)	
Partially Opposed	21 (1.6)	0.93 (0.70, 1.25)		8 (2.2)	0.95 (0.60, 1.51)	
Partially in Favor	162 (12.3)	0.86 (0.73, 1.02)		41 (11.5)	0.71 (0.45, 1.14)	
Completely in Favor	964 (73.1)	0.96 (0.85, 1.08)		269 (75.6)	0.79 (0.59, 1.06)	
PARTNERS	***N*** **= 444**			***N*** **= 121**		
*Transactional encounter with any of last 3 partners* [ref.: no]	60 (13.5)	0.94 (0.80, 1.10)	0.95 (0.81, 1.12)	55 (45.5)	0.92 (0.70, 1.22)	0.96 (0.73, 1.26)
*Alcohol used with any of last 3 partners by partner or participant prior to sex* [ref.: no]	232 (52.3)	**1.18 (1.07, 1.31)**	**1.13 (1.02, 1.26)**	70 (57.9)	1.18 (0.45, 0.72)	1.12 (0.83, 1.51)

***Abbreviations*:** *aPR* adjusted prevalence ratio, *PR* prevalence ratio, *95% CI* 95% confidence interval

Notes: *Ns* for individual variables may vary, depending on completeness of participant responses. For variables missing responses, Ns are as written

Bold values indicate *p* < 0.05 in crude (bivariate) and adjusted (multivariate) generalized estimating equation (GEE) models

Multivariate GEE models were adjusted for age, sexual role, sexual orientation, education, partnership type, and alcohol use prior to sex

^a^recategorized to: “family, partner, friend, or colleague” prior to inclusion in GEE.

^b^recategorized to: “never or ever” prior to inclusion in GEE.

Among the 363 close social network members described by trans women, 28.2% were perceived to identify as heterosexual; 25.9%, as bisexual; 24.7% as transgender; and 21.2% as gay. Trans women reported “never” discussing HIV/STI prevention with 30.0% of SNMs. Discussions were least common with cis-gender, heterosexual-identified SNMs (40.2% “never” discussed) and most common with transgender SNMs (27.1% “at least once weekly”). Trans women believed most SNMs to be completely in favor of condom use (61.5%) and HIV/STI testing (75.6%), but described less support for partner notification of an STI diagnosis (37.4% completely in favor). Colleagues were perceived as least supportive of condom use (7.7% completely opposed), and sexual partners, as most supportive (28.0% completely in favor). Among trans women, 62.8% reported CAI with any of their previous 3 partners.

No SNM attitudes or characteristics were significantly associated with CAI among MSM or trans women.

## Discussion

Our analysis provides important information about the close social networks, peer norms, and sexual practices of Peruvian MSM and trans women. Although participants perceived strong social support for condom use by SNMs, they also reported frequent CAI. While the composition of their social networks and SNM relationships were fundamentally distinct, both MSM and trans women populations reported similar perceptions of SNM support for condom use, HIV/STI testing, and partner STI notification. These findings suggest potential overlap in perceived sexual norms between MSM and trans women’s communities while also reinforcing the need to tailor outreach and prevention efforts to the distinct social and sexual contexts of these two groups [16].

Expanding upon prior Peruvian literature, we identified differences in MSM and trans women’s overall social and sexual network characteristics as well as in SNM identity and discussion patterns [27, 28]. Trans women reported larger social and sexual networks, greater heterogeneity in SNM sexual orientation and gender identity, and increased involvement in transactional sex compared to MSM. Both groups preferentially discussed HIV and STI prevention with SNMs whose sexual orientations and gender identities aligned with their own: Among MSM, discussions were most frequent with SNMs from the gay and bisexual community, while trans women communicated most with others perceived as transgender. These results signal the importance of homophily in discussions about HIV and STIs and highlight the role of peers as sources of sexual health information [15, 16]. However, as peer discussions often address HIV/STI risk only superficially, greater attention toward the content and impact of community-level sexual health communication is warranted [15, 16].

We found that both MSM and trans women perceived less social support for partner notification of an STI diagnosis (33.4% MSM SNM, 37.4% trans women SNM “completely in favor”) compared to other sexual health behaviors. This result may be situated within prior Peruvian literature, as previous work has found partner notification practices to vary with factors such as partnership type (in relation to perceived risks and benefits of notification as well as ability to contact partners), type of STI diagnosed (HIV or other STIs), perception of peer support for notification, and fear of rejection or interpersonal violence [29–31]. It is notable that participants reported low levels of HIV serostatus communication with sexual partners in a separate analysis from this same study cohort, as our finding regarding social support for partner STI notification may reflect broader reticence in sexual communication norms [23].

We did not observe an association between close SNM attitudes or characteristics and participant-reported CAI. While prior studies have signaled a role for social network structures and norms in condom use decisions [1, 3, 7, 14], a potential explanation for the negative finding in our study may be the importance of structural, situational, and dyadic characteristics in shaping sexual practices of MSM and trans women [16, 17]. For example, we found that alcohol use was associated with CAI among MSM, adding to an existing body of literature from Latin America describing a relationship between alcohol consumption and condomless intercourse among MSM and TW [32–34]. In addition, our study examined perceived norms surrounding participants’ use of condoms. In a study of young African American MSM in the United States, Peterson et al. noted discordances in participant dyads’ perceptions of peers’ sexual practices and attitudes toward their own; while most dyads reported that SNMs approved of their use of condoms, they were less likely to think the same SNMs were using condoms themselves [2]. Research from the Dominican Republic found that peer encouragement to use condoms was not associated with consistent condom use among men, suggesting potential limitations to perceived or stated peer approval of sexual health behaviors [20]. Given previous evidence of “risk clustering” within social networks, and discordances between publicly articulated knowledge of health promotion practices and commonly understood vernaculars of sexual behavior, further study should explore the interplay of public health standards with actual community norms, and their implications for HIV and STI transmission [1, 2, 35].

Lastly, our data showed an association between educational attainment (> high school education) and CAI among trans women. This result differs from previous research findings, as higher educational attainment has typically been shown to be associated with condom use and other safer sexual practices in multiple global contexts [36–38]. The relationship we observed may have been influenced by factors not captured by our study and points to a need for continued research into the impact of education and other socio-structural factors on HIV and STI risk among this population [39].

Several limitations exist to this study. As data was collected by self-report, social desirability bias may have influenced participant reports of sexual norms and practices. There may also be inaccuracies in SNM characteristics, due to variability between participants’ interpretations of questions and responses. Overlap in social and sexual relationships may have blurred the boundaries between patterns of discussion with SNMs and high-risk behavior with sexual partners [5]. Finally, as we recruited a convenience sample of behaviorally high-risk MSM and trans women, and the parent study was not designed to explore relationships between SNM characteristics and sexual behavior, norms and behaviors may not be generalizable outside of these groups.

## Conclusion

By characterizing close social contacts of Peruvian MSM and trans women, our analysis provides important information about social structure and influence within these communities, guiding outreach and prevention efforts. Future research should explore how to understand and address sexual health norms in the social and structural contexts experienced by these populations.

## Data Availability

The datasets generated and/or analysed during the current study are not publicly available due to restrictions on the publication of data without participant consent, but are available from the corresponding author on reasonable request and with approval from the IRBs.

## Abbreviations

aPR: Adjusted prevalence ratio
CAI: Condomless anal intercourse
CI: Confidence interval
CRAI: Condomless receptive anal intercourse
GEE: Generalized estimating eq.
HIV: Human immunodeficiency virus
IQR: Interquartile range
LGBT: Lesbian, gay, bisexual, and transgender
MSM: Self-identified men who have sex with men
PR: Prevalence ratio
SNM: Social network member
STI: Sexually transmitted infection
Trans women: Self-identified transgender women
